# A study on the impact of gargling with compound *Scutellaria baicalensis* Georgi on oral health and microflora changes in fixed orthodontic patients: An experimental study

**DOI:** 10.1097/MD.0000000000039397

**Published:** 2024-08-23

**Authors:** Peng Zhang, Shen Guo Chen, Jia Ting Wang, Jin Dong Wang, Zai Hong Chen, Hai Sheng Lin

**Affiliations:** a Taizhou Hospital of Zhejiang Province affiliated to Wenzhou Medical University, Lin Hai, Zhejiang, China; b Enze Hospital, Taizhou Enze Medical Center (Group), Taizhou, Zhejiang, China.

**Keywords:** compound *Scutellaria baicalensis* Georgi gargle, fixed orthodontics, oral flora, periodontal health

## Abstract

**Purpose:**

To investigate the effect of *Scutellaria baicalensis* Georgi gargle on oral health and changes in oral bacteria among orthodontic patients.

**Methods:**

About 110 cases of oral fixed orthodontic patients were screened from January 2020 to June 2022 at Taizhou Hospital in Zhejiang Province. They were randomly divided into the experimental group (receiving compound *S. baicalensis* Georgi gargle once a day) and the control group (receiving 0.9% NS gargle once a day), with 55 cases in each group. Gingival samples were collected from both groups before and 3 months after the orthodontic surgery for bacterial culture, and the differences between the 2 groups of patients in Plaque Index (PLI), gingival bleeding index (sBl), and periodontal depth (PD) before and after the operation were compared. Results: The detection levels of PLI, PD, and sBI in the experimental group were lower than those in the control group (*P* < .05) 3 months after orthodontic surgery (*P* < .05); after orthodontic correction for 3 months, there was a significant difference in coccus, bacillus, Campylobacter, Clostridium, Helicobacter, and filamentous bacteria between the experimental group and the control group (*P* < .05); and *Porphyromonas gingivalis*, *Fusobacterium nucleatum*, *Bacteroides forsythus* (B.f), and Agglomerata actinomycetes in the 2 groups were statistically significant after 3 months of orthodontic treatment (*P* < .05).

**Conclusion subsections:**

In fixed orthodontic treatment, *S. baicalensis* Georgi gargle can effectively inhibit oral pathogens and maintain periodontal health.

## 1. Background

With the continuous progress of society, people are gradually putting a new premium on oral problems. A growing number of patients with malocclusion are opting for orthodontic treatment at the Department of Stomatology, while most adults opt for fixed orthodontics. However, in the process of fixed orthodontics, appliance attachments often need to be bonded to the tooth surface, which will affect the cleaning of the tooth surface and the self-cleaning effect of the oral cavity.^[1]^ What’s more, in the initial stage of appliance wearing or after orthodontic force, varying degrees of acid swelling and pain, as well as periodontal tissue sensitivity, will appear in the local or whole teeth, which will also affect the cleaning of teeth, resulting in food residue remaining around the attachment and plaque accumulation. In severe cases, this may lead to chronic inflammation of the gums, demineralization of the tooth surface, caries, etc.^[1,2]^ Therefore, proper oral hygiene maintenance is particularly important. In clinical practice, compound chlorhexidine gargle is also used to control and treat gingivitis. However, this gargle is not recommended for long-term use because it has a slightly bitter taste, and may also cause tooth coloring, transient loss of taste, blackening of the back of the tongue and allergic reaction, etc. Nowadays, gradually stricter clinical control has been proposed for western antibacterial drugs, so natural drug gargle has attracted more and more attention from clinicians.^[3,4]^ In this paper, a compound gargle of *Scutellaria baicalensis* Georgi is proposed to inhibit oral pathogens. *S. baicalensis* Georgi has been shown in some studies to have the effect of effectively inhibiting lipopolysaccharide of pathogenic bacteria,^[5,6]^ while baicalin is believed by some scholars to effectively promote collagen synthesis and proliferation of endothelial cells.^[7]^ At present, few studies have been carried out on the clinical effect of the compound gargle of *S. baicalensis* Georgi. In view of this, in this paper, we mainly analyzed the oral hygiene status of patients with fixed orthodontics and the changes of bacterial flora in the gingival sulcus before and after treatment with or without the use of compound gargle of *S. baicalensis* Georgi. The specific findings are as follows.

## 2. Subjects and methods

A fully randomized design was employed, with a necessary sample size of 55 patients for each group. A total of 110 patients (aged between 18 and 35 years) with fixed orthodontics admitted to Taizhou Hospital of Zhejiang Province from January 2020 to June 2022 were randomly divided into 2 groups: the experimental group (gargling with a compound gargle of *S. baicalensis* Georgi once a day) and the control group (gargling with normal saline once a day), with 55 patients in each group. The inclusion criteria were as follows: Adequate compliance and proper brushing, as well as oral hygiene maintenance following corrective treatment; Plaque Index (PLI) ≤ 20% prior to orthodontic intervention; and periodontal tissue health and complete dentition in both upper and lower jaws prior to orthodontic treatment.^[8]^ All patients voluntarily participated and signed informed consent forms. The hospital ethics committee approved the study protocol prior to implementation.

### 2.1. Sample collection and bacterial culture

Bacteria in the gingival sulcus of the patients were collected before and 3 months after the operation. First, the dental plaque was removed, the tissue samples in the gingival groove were absorbed with sterile paper and placed in a sterile test tube after the tooth surface was dried, and 1mL of sterile PBS solution was added and stored at −80 °C for later use. Subsequently, the collected samples were inoculated with tryptone soybean broth medium (TSB, Thermo, Shang Hai, China, CM0129B) and brain–heart infusion blood AGAR plate (Thermo, Shang Hai, China, CM1136B), respectively, and placed in a 37 °C constant-temperature incubator for 48 h of anaerobic culture. An automatic bacterial tester (Thermo Fisher Scientific, Shang Hai, China) was used for bacterial detection and identification. The detection results were in accordance with the 2010 (M100–S20) standards of the Clinical and Laboratory Standards Institute (CLSI).

### 2.2. Observation index records

PLI including the number and thickness of plaques – 0: sterile plaque; 1: a small amount of plaque; 2: plaque covering ≤ 1/3 of the tooth surface; 3: plaque covering ≥ 1/3 of the tooth surface. Sulcus bleeding index (sBI) – 0: normal; 1: mild gingival edema without bleeding; 2: gingival edema and bleeding; 3: spontaneous bleeding and ulcer of the gums. Periodontal probing depth (PD) was measured in the distal, central, and proximal parts of the buccal and lingual sides of the right lower first molar, and a maximum value of 1 mm was obtained.^[8]^ Changes in PLI, sBI, and PD in the fixation correction group before surgery and 3 months after surgery were compared with those in the control group.

### 2.3. Statistical analysis

All data in this study were analyzed using SPSS 20.0. Statistical differences among groups were tested using the normal distribution test, the Wilcoxon rank-sum test, and the rates among groups were compared using the χ^2^ test or Fisher exact probability test. Statistical significance was set at *P* < .05.

## 3. Results

### 3.1. Comparison of the differences in clinical indexes of periodontal conditions

**Figure 1.**
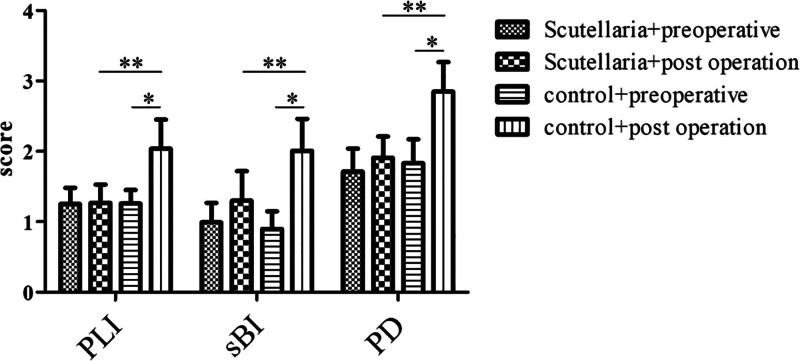
Changes in periodontal clinical indexes between the orthodontic group and the control group at different times (*P* < .05).

No significant difference was observed in PLI, sBI, and PD between the *S. baicalensis* Georgi and control groups before orthodontic treatment (*P* > .05). The levels of PLI, PD, and sBI were lower than those in the control group at 3 months after orthodontic treatment (all *P* < .05), while the levels of PLI, PD, and sBI in the control group were significantly higher than those before and after orthodontic treatment (all *P* < .05), as shown in Figure 1.

### 3.2. Comparison of bacterial distribution in gingival crevicular fluid

No significant differences were observed in the identification of cocci, bacilli, fusiform bacteria, spirillum, Campylobacter, and filamentous bacteria in the gingival crevicular fluid between the *S. baicalensis* Georgi and control groups before surgery (*P* > .05). The changes in cocci, bacilli, fusiform bacteria, spirillum, Campylobacter, and filamentous bacteria in the gingival crevicular fluid in the compound *S. baicalensis* Georgi group before and after orthodontic treatment were not statistically significant (*P* > .05). In the control group, the proportion of cocci, bacilli, and fusiform bacteria in the gingival crevicular fluid decreased significantly before and after 3 months of orthodontic treatment, whereas the proportions of spirillum, Campylobacter, and filamentous bacteria increased significantly (*P* < .05). Compared with the control group, the number of cocci, bacilli, and Campylobacter in the compound *S. baicalensis* Georgi group increased after 3 months of orthodontic treatment, while the proportion of fusiform bacteria, spirochetes, and filamentous bacteria decreased significantly (*P* < .05), as shown in Table 1.

**Table 1:** Changes of bacteria distribution in the gingival crevicular fluid between the orthodontic group and the control group at different times ($\bar{x}\pm s$).

Group	Time	Cocci	Bacilli	Campylobacter	Fusiform bacteria	Spirillum	Filamentous bacteria
Scutellaria (n = 55)	Preoperative	57.2 ± 2.4	35.3 ± 1.8	2.9 ± 0.3	4.7 ± 0.3	3.1 ± 0.4	2.9 ± 0.9
	Post operation	50.8 ± 3.2	33.2 ± 2.2	2.1 ± 0.2	4.1 ± 0.4	2.9 ± 0.5	2.4 ± 1.2
Control (n = 55)	Preoperative	54.5 ± 3.6	34.9 ± 1.9	3.2 ± 0.5	4.8 ± 0.6	3.5 ± 0.3	3.2 ± 1.1
	Post operation	42.6 ± 4.6*	22.7 ± 4.8*	1.4 ± 0.4*	21.1 ± 1.8*	4.2 ± 0.3*	19.8 ± 2.4

*P < 0.05.

### 3.3. Detection of main pathogenic bacteria in gingival crevicular fluid

No statistically significant difference was observed in the detection rates of *Porphyromonas gingivalis*, *Fusobacterium nucleatum*, *Bacteroides forsythus* and *Agglomerata actinomycetes* in the gingival crevicular fluid between the compound *S. baicalensis* Georgi group and the control group before surgery (*P* > .05). After 3 months of orthodontic treatment, the detection rate of the above 4 bacteria in the compound *S. baicalensis* Georgi group was significantly lower than that in the control group (*P* < .05), and the 4 bacteria in the control group were significantly increased before and after surgery (*P* < .05), as shown in Table 2.

**Table 2:** Comparison of bacteria detection in the gingival crevicular fluid between the orthodontic group and the control group at different times.

Group	Time	*P. gingivalis*		*F. nucleatum*		B.f		*A. actinomycetes*	
		Quantity	Detection rate, %	Quantity	Detection rate, %	Quantity	Detection rate, %	Quantity	Detection rate, %
Scutellaria (n = 55)	Preoperative	3	5.45	9	16.36	4	7.27	10	18.18
	3 months	1	1.82	10	18.18	3	5.45	9	16.36
Control (n = 55)	Preoperative	4	7.27	8	14.55	5	9.09	11	20.00
	3 months	13	23.64*	27	49.09*	29	52.73*	31	56.36*

*P < 0.05.

## 4. Discussion and conclusions

Currently, fixed orthodontics is preferred as a means to treat malocclusion. Patients undergoing fixed orthodontics often need to wear fixed orthodontics such as metal brackets and orthodontic arch wires, resulting in easy accumulation of food residues on the teeth, and the surface of orthodontics is not easy to clean. As a result, unfavorable consequences such as plaque accumulation, gingival inflammation, tooth demineralization, tooth decay, and the effect of orthodontic correction^[9]^ may also be affected, resulting in the accumulation of a large number of acid-producing and acid-resistant caries-causing bacteria on the tooth surface.^[10,11]^ *S. baicalensis* is the main active ingredient of the compound *S. baicalensis* Georgi, boasting many functions such as antibacterial, heat-clearing, dampness-drying, detoxification, and antiallergic reactions. Its specific molecule, baicalin, can inhibit the formation of inflammatory factors and has antiinflammatory effects.^[12]^ Baicalin may directly promote the proliferation of human periodontal ligament fibroblasts and inhibit the secretion of tumor necrosis factor-α (TNF-α) in periodontal ligament cells by degrading the endotoxin lipopolysaccharide (LPS) of pathogenic bacteria.^[7,13]^ It has been reported in the literature that the combination of *S. baicalensis* Georgi gargle after basic periodontal treatment can significantly relieve the periodontal symptoms of patients with diabetes, and it has been suggested that the mechanism may be that *S. baicalensis* Georgi gargle can effectively reduce inflammatory markers and oxidative stress products. Recent animal experiments have shown that baicalein can also reduce the phosphorylation level of NF-κB p65 protein in rat gingival fibroblasts induced by LPS, reduce the damage to alveolar bone and gingival fibers, and effectively inhibit the absorption of alveolar bone, thus alleviating periodontal symptoms.^[14]^

As suggested by the results of this study, there was no significant difference in PLI, sBI, and PD levels between the experimental and control groups before orthodontic treatment (*P* > .05), indicating that the oral hygiene conditions of the patients before treatment were basically consistent. After 3 months of orthodontic treatment, the levels of PLI, sBI, and PD in the compound *S. baicalensis* Georgi group were significantly different from those in the control group (*P* < .05). Compared with the control group before and after orthodontic treatment, the detection levels of PLI, PD, and sBI increased significantly (all *P* < .05), indicating that regular use of A. membranaceus in patients with fixed orthodontics could effectively maintain oral hygiene.

More than 300 kinds of bacteria have been reported in the gingival sulcus, of which approximately 90% are anaerobic bacteria that are closely related to periodontal health.^[15]^ The change in gingival sulcus fluid also has a close bearing on the development of periodontal disease.^[16]^ As shown in the results of this study, there was no statistically significant difference in the identification of cocci, bacilli, fusiform bacteria, spirillum, Campylobacter, and filamentous bacteria in the gingival crevicular fluid between the compound *S. baicalensis* Georgi group and the control group before surgery (*P* > .05). The changes in cocci, bacilli, fusiform bacteria, spirillum, Campylobacter, and filamentous bacteria in the gingival crevicular fluid in the compound *S. baicalensis* Georgi group before and after orthodontic treatment were not statistically significant (*P* > .05). In the control group, the proportion of cocci, bacilli, and fusiform bacteria in the gingival crevicular fluid decreased significantly before and after orthodontic treatment, whereas the proportions of spirillum, Campylobacter, and filamentous bacteria increased significantly (*P* < .05). Compared with the control group, the number of cocci, bacilli, and Campylobacter in the compound *S. baicalensis* Georgi group increased after orthodontic treatment, whereas the proportion of fusiform, spirochete, and filamentous bacteria decreased significantly (*P* < .05).The reason may be that Clostridium is an anaerobic bacterium, while Spirillum and filamentous bacteria are anaerobic bacteria. Both are suitable for survival and proliferation in the low-oxygen environment of the posterior gingival sulcus, leading to gingivitis and periodontitis.^[17,18]^ However, the mouths of most normal people are dominated by cocci and bacilli. After the healthy periodontal environment is destroyed, the number of cocci and bacilli decreases accordingly. If oral hygiene is maintained through the use of chlorhexidine, the oral hygiene environment can be significantly improved. Therefore, in the control group, the number of cocci, bacilli, etc decreased significantly 3 months after operation (*P* < .05), indicating that after using compound astragalus membranaceus, the normal flora structure of the oral cavity was maintained, and the periodontal health of oral cavity could be effectively maintained.

It is currently believed that *P. gingivalis*, *F. nucleatum*, B.*forsythus*, and *A. actinomycetes*, as key pathogenic bacteria of periodontitis, are closely related to the occurrence of periodontitis.^[19]^ Some experiments have shown that baicalin has an inhibitory effect on periodontal pathogens (e.g., *F. nucleatum*, B.*forsythus*, and *A. actinomycetes*).^[20]^ In this study, no significant difference was observed in *P. gingivalis*, *F. nucleatum*, B.f, and *A. actinomycetes* between the compound *S. baicalensis* Georgi group and the control group before orthodontic treatment (*P* > .05). After 3 months of orthodontic treatment, the 4 periodontal pathogens in the compound *S. baicalensis* Georgi group decreased significantly compared with those before orthodontic treatment (all *P* < .05) and also decreased significantly compared with the control group (*P* < .05). This shows that *S. baicalensis* Georgi can inhibit periodontal pathogens in the gingival sulcus and prevent periodontal disease. After 3 months of orthodontic treatment, the detection rate of these 4 periodontal pathogens increased compared to that before orthodontic treatment (*P* < .05), which could be attributed to the fact that periodontal pathogens were easily attached to the tooth surface and gingival sulcus due to the difficulty of maintaining oral hygiene during orthodontic treatment. This further shows that patients undergoing fixed orthodontic treatment can effectively inhibit the growth and reproduction of periodontal pathogens after regular use of the compound *S. baicalensis* Georgi. Hence, this mouthwash can be utilized for the prevention of dental caries and periodontal disease in patients undergoing orthodontic treatment. Moreover it could be interesting in the future to test *S. baicalensis* Georgi gargle in combination with other recently introduced preventive treatments such as Ozone,^[21]^ photobiomodulation^[22]^ and remineralizing pastes^[23]^ in order to understand their mutual effect on oral microbiota.

In summary, fixed orthodontics have a certain influence on oral flora, and the number of periodontal pathogens also increases. Regular use of the compound *S. baicalensis* Georgi can effectively inhibit oral pathogens and prevent periodontal disease. It is necessary for patients undergoing fixed orthodontic treatment to maintain oral hygiene using an antibacterial gargle regularly.

## Author contributions

**Data curation:** Shen Guo Chen.

**Funding acquisition:** Jin Dong Wang.

**Investigation:** Jin Dong Wang.

**Methodology:** Shen Guo Chen, Zai Hong Chen.

**Resources:** Zai Hong Chen.

**Supervision:** Zai Hong Chen, Hai Sheng Lin.

**Validation:** Jia Ting Wang.

**Writing – original draft:** Peng Zhang, Jia Ting Wang.

**Writing – review & editing:** Peng Zhang, Hai Sheng Lin.
